# Clinical progression of renal vein leiomyoma: A case report

**DOI:** 10.1016/j.ijscr.2019.10.067

**Published:** 2019-11-04

**Authors:** Kuldeep Dhawan, Nakul Bansal, N.M. Gupta, Simran Dhawan

**Affiliations:** Dhawan Hospital, Plot #1, Sector 7, Panchkula, Haryana, 134109, India

**Keywords:** CT, computed tomography, GFR, glomerular filtration rate, SCARE, Surgical Case Report Guidelines, CECT, contrast-enhanced computed tomography, IVC, inferior vena cava, NSAIDs, nonsteroidal anti-inflammatory drugs, ER, estrogen receptor, PR, progesterone receptor, Ki-67, a nuclear protein used as a cellular marker for proliferation, FNA, fine needle aspiration, Leiomyoma, Renal vein, Angioleiomyoma, Vascular leiomyoma, Flank pain, Case report

## Abstract

**Introduction:**

Vascular leiomyoma originating in the renal vein is a rare entity. Our case allowed us charting of progression of renal vein leiomyoma for 8-years’ duration. Apart from intraluminous growth causing displacement of viscera, the leiomyoma did not show any aggressive behaviour. Eventually, removal of the tumour through nephrectomy provided complete symptomatic relief to our patient.

**Case presentation:**

A 75-year old female presented with an eight-year long history of severe pain in right flank. CT imaging revealed a mass arising from the right renal vein and a few hepatic tumours. The differentials included leiomyosarcoma, in view of hepatic lesions. Excision of the tumour was performed with radical nephrectomy. Immunohistopathological examination confirmed it to be a case of leiomyoma. Three months post-op, the patient reported complete pain relief and no adverse events, and there were no signs of recurrence.

**Discussion and conclusion:**

Vascular leiomyomas do not show any aggressive behaviour. However, their growth tends to displace the normal viscera, causing pressure effects that may give rise to intractable pain. Vascular obstruction affects GFR and renal function. In our patient, despite a long period of growth, the tumour did not show any malignant changes. Tumour excision through radical nephrectomy resulted in symptomatic improvement in our patient. Since imaging alone does not distinguish a leiomyoma from its malignant counterpart with certainty, surgical excision may be warranted in all the cases.

## Highlights

• Vascular smooth muscle tumours are rare entities. Majority of these are malignant.
• Differentiation between a vascular – leiomyoma and a leiomyosarcoma is chiefly upon histopathological examination. Imaging alone is non-contributory.
• The rarity of the tumour makes predicting the prognosis a challenging prospect.
• Renal Leiomyomas do not show aggressive behaviour and excellent prognosis is predicted post nephrectomy.
• An eight-year long charting of progress of leiomyoma in our case has demonstrated the non-malignant potential of such tumours.

## 1 Introduction

While leiomyomas can occur in any organ that contains smooth muscle, they are more common in the uterus [1]. Vascular leiomyomas are of uncommon occurrence. Even in the genitourinary tract, leiomyomas most commonly arise in the renal capsule [2,3]. The inability of imaging to distinguish the vascular leiomyoma from its malignant counterpart, as evidenced in literature [4], proved to be the major diagnostic challenge. We are reporting one such case of a Renal Vein Leiomyoma extending into the inferior vena cava, in line with the SCARE criteria [5].

## 2 Case report

A 75-year old homemaker presented to us in November 2018 with severe pain in right flank of eight years duration. On examination, she was moderately obese (weight: 68 kg; height: 156 cm); the general physical exam was essentially normal and abdominal physical exam did not reveal any abnormality. She reported a history of diabetes mellitus and hypertension.

After a gradual onset, 8 years ago, the intensity of her pain stayed 2–3 on a 10-point scale for the next 4–5 years. The pain had no aggravating factor and was relieved with meals. It was dull aching in quality and non-radiating. It did not interfere with her daily activities and rarely woke her up from sleep at night. The pain episodes always occurred at fixed time of the day. She had 1–2 such episodes per day on an average. A complaint of persistent bilateral pedal oedema unamenable to limb elevation and tight stockings was also elicited. Imaging done at that time had suggested of a renal tumour and a biopsy was scheduled. The patient declined any further treatment from then on. She sought pain relief from Homeopathic medication.

**Fig. 1:**
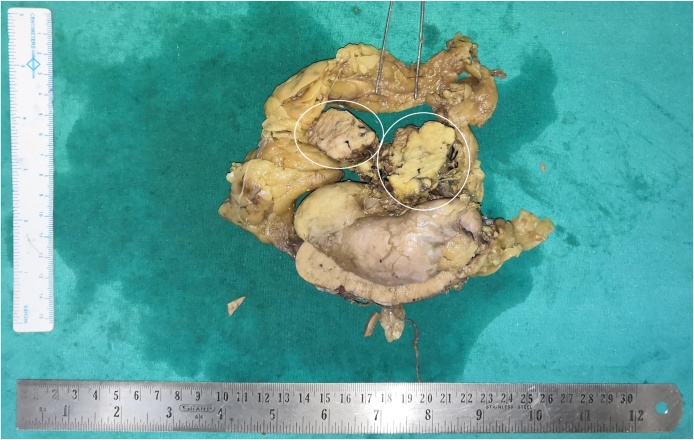
The post nephrectomy gross specimen of the resected right kidney shows a firm and well circumscribed tumour (encircled) originating from the renal pedicle and displacing the renal vein.

**Fig. 2:**
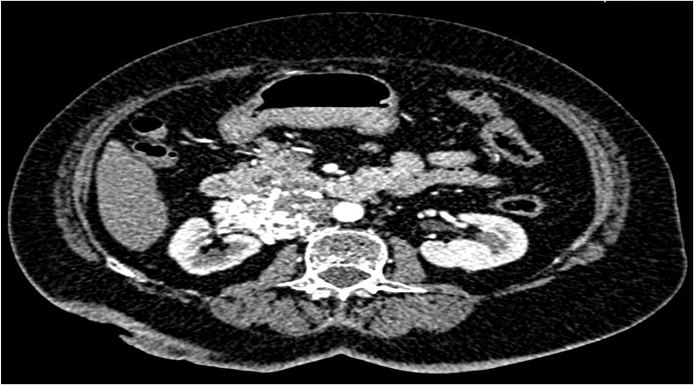
Transverse section CECT abdomen showing a tumour of size 6.3 × 4.1 × 4.7 cm originating from right renal vein and extending into inferior vena cava.

**Fig. 3:**
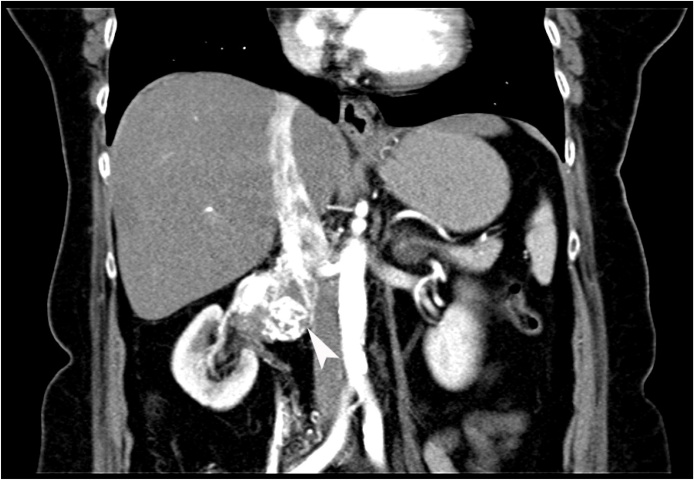
Coronal section CECT abdomen showing a tumour of size 6.3 × 4.1 × 4.7 cm originating from right renal vein and extending into inferior vena cava (white arrowhead).

**Fig. 4:**
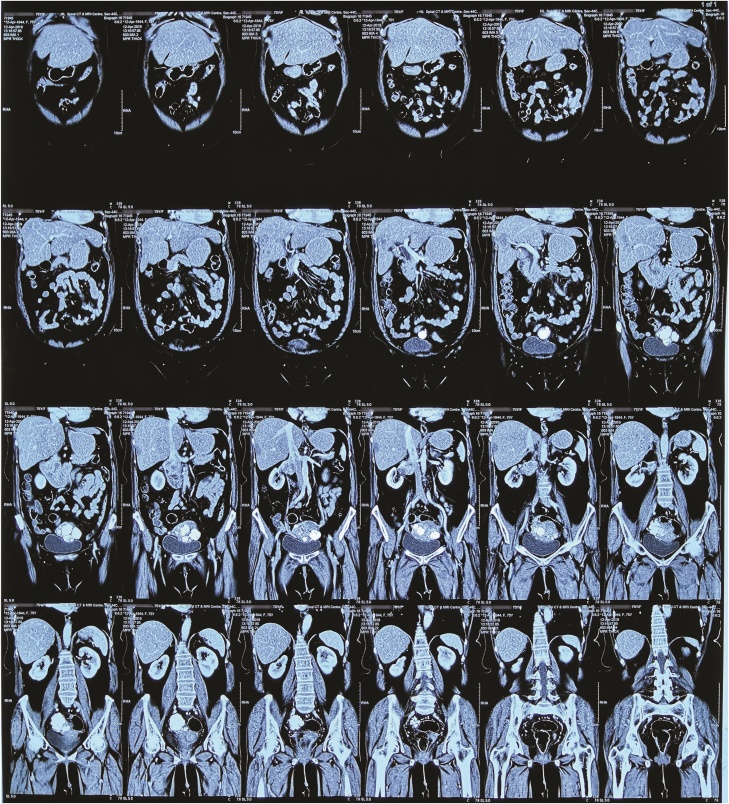
Coronal section images of abdominal CECT showing the tumour originating from Right Renal Vein and extending into the Inferior Vena Cava.

Six years ago, she underwent open cholecystectomy for symptomatic cholelithiasis. However, a month of pain-free period post cholecystectomy was followed by a return of pain of same intensity and character (Fig. 1, Fig. 2, Fig. 3, Fig. 4).

For the last couple of years, patient reported worsening of pain. She rated the pain intensity at 10/10. The pain evolved more constant frequency and began radiating to her back.

During her visit in November 2018, she had declined further imaging and had opted for continuation of palliative care instead.

Later, a CECT done in April 2019 showed a 6.3 × 4.1 × 4.7 cm dense space occupying lesion in right paracaval retroperitoneal region. This was a significant increase in the dimensions of the tumour from the last imaging done a year ago that had measured it at 3.12 × 4.2 × 3.6 cm. The growth was splaying the renal vein and had advanced through inferior vena cava’s (IVC) lumen, causing its narrowing. There were prominent soft tissue densities in lymph nodes in peripancreatic retroperitoneal region. There were 2 focal hepatic mass lesions measuring 3 × 3.6 × 3.2 cm and 1.5 × 3 × 2.5 cm in subcapsular location. Note was also made on presence of multiple uterine leiomyomata. A tentative diagnosis of metastatic leiomyosarcoma of the right kidney was made. By this time, she still had significant residual pain despite medication with maximal doses of Dicyclomine, NSAIDs and opioids. At this visit, the patient agreed to a palliative surgical resection of the tumour.

During surgery, a large hypervascular lobulated lesion was discovered medial to the right kidney, inseparably adherent to the right renal hilum, encasing and splaying the right renal vein with intraluminal extension into the infrahepatic IVC. Extensive colonic and omental adhesions required careful dissection. Further up, the growth was adherent to the 2nd–3rd part of duodenum and the uncinate process of the head of pancreas, without any infiltration. The growth was encroaching upon abdominal aorta at the level of right renal hilum, which needed to be carefully dissected out. An en-bloc right nephrectomy was performed to remove the renal lesion. The growth in the IVC required a 10 cm excision of its lateral wall and reconstruction. The post-op course was uneventful and the patient made a full recovery from the surgery.

**Fig. 5:**
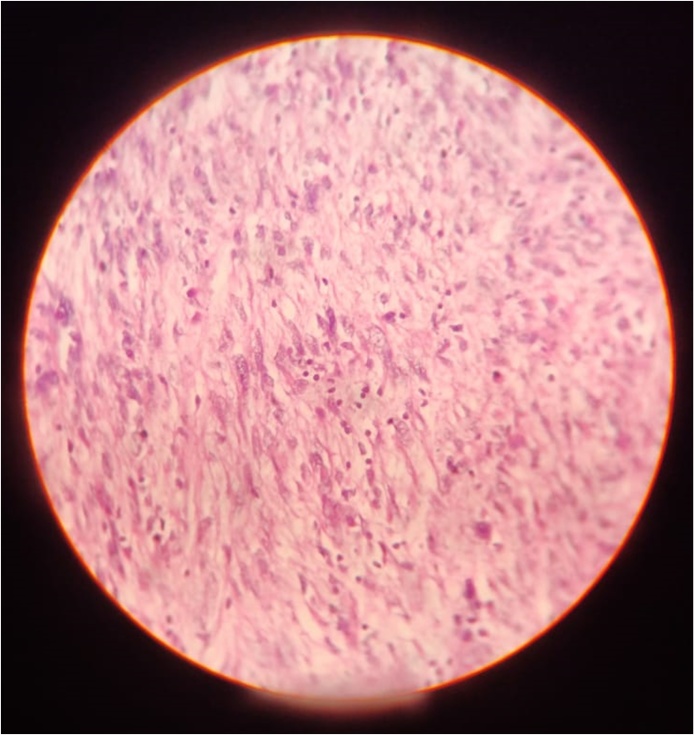
40× magnification | haematoxylin and Eosin stained section. Image shows interlacing bundles of smooth muscle cells in well vascularised stroma. No atypical mitotic figures seen. Multiple sections examined that showed minimal inflammatory infiltrate.

**Fig. 6:**
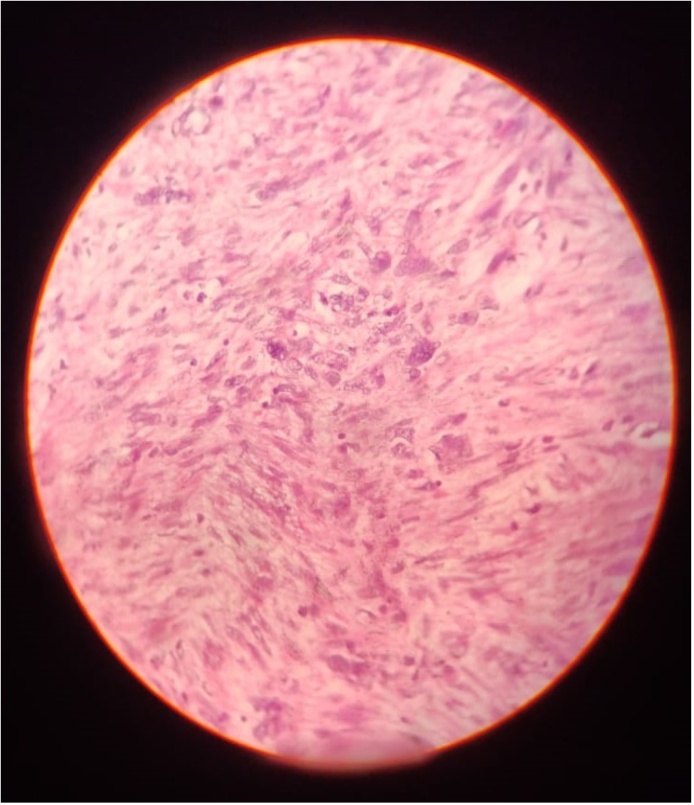
40× magnification | Haematoxylin and Eosin stain. Image shows high cellularity. Occasional mitotic activity seen. Atypical mitotic figures not seen.

The report on the histopathological sample of the resected growth (Fig. 5, Fig. 6) showed: interlacing bundles of smooth muscle cells placed in well vascularised stroma. Few mitotic figures were seen, but without any atypical mitosis. Immunofluorescence results for the tumour were negative for ER and PR, showed low Ki-67 index and returned positive for H-Caldesmon. The report confirmed the tumour as a venous leiomyoma. The hepatic lesions proved to be benign upon fine needle aspiration (FNA) cytological examination.

Three months after the surgery, the patient reported near complete absence of the debilitating pain. She experienced 1–2 episodes of pain rated at 3–4/10 in intensity occurring at fixed time of the day, associated with an onset of urge to defecate and completely relieved by defecation or when the urge to defecate died down. A repeat CT scan of abdomen done in October, 2019, did not reveal any evidence of disease recurrence.

## 3 Discussion

Leiomyomas are benign mesenchymal tumours that commonly arise in smooth muscle–lined hollow organs. Histologically, leiomyomas may be classified as either simple, epithelioid, or with a prominent vascular component (vascular leiomyoma or angiomyoma). Vascular leiomyomas are further classified as capillary angioleiomyomas, cavernous type or venous angioleiomyomas [6]. Among a few hundred cases of venous smooth muscle tumours described in literature, majority are leiomyosarcomas that originate in the Vena Cava [7]. Currently, the differential diagnosis between leiomyoma and leiomyosarcoma is only histopathological after surgical resection. Extensive central necrosis, invasive growth, and a heterogeneous appearance are suggestive of leiomyosarcoma. However, it is not possible to differentiate a leiomyoma from leiomyosarcoma on the basis of imaging alone [4]. Upon microscopic examination, leiomyomas have fusocellular elements showing the absence of mitotic figures, pleomorphism, hypercromatism and, above all, an absence of perilesional invasivity [2].

The Leiomyomas tend to grow from their origin in the wall of the vein, displacing the nearby structures, rather than infiltrating them. But they can grow along the vein wall and spread within its lumen in a downstream direction which can lead to thrombosis. Hence, the recommended procedure is excision of the mass and ligation of the feeder vessels [7,8].

Upon extensive search of literature, only five cases of leiomyoma of renal vein were found to have been reported [1,7,[9], [10], [11]]. The displacement of viscera by the growing tumour is likely responsible for the intractable pain experienced by these patients. Renal afferent nerve supply travels along sympathetic fibres to end in T11 – L2 dorsal spinal nerve roots, from where the pain is referred to the corresponding dermatomes in the flank [12]. While one case had an incidental detection of the tumour, four others had a history of acute onset of pain that prompted further investigations, followed by surgical removal. Our case allows us the documentation of clinical progression of a venous leiomyoma of 8 years duration. Our patient finally had relief of her pain after surgical excision of the tumour.

## 4 Conclusion

We reported a rare case of renal vein leiomyoma that clinically presented with flank pain of long duration. Eventually, tumour excision, along with nephrectomy, provided symptomatic relief to the patient. There were also no signs of tumour recurrence, just as reported in the literature. The diagnostic challenges and the pre-op clinical progression of the disease makes our case unique.

## Funding

This report did not receive any kind of funding or sponsorship from any external agency.

## Ethical approval

The hospital ethics committee verified that the patient’s treatment did not involve any experimental procedure and all standard of care procedures were followed. This case report manuscript was approved by the ethics committee. The approval code was DHE401.

## Consent

Written informed consent was obtained from the patient for publication of this case report and accompanying images. A copy of the written consent is available for review by the Editor-in-Chief of this journal upon request.

All non-essential details have been omitted to maintain patient’s privacy and to ensure anonymity.

## Author contribution

All doctors were directly in charge of medical and surgical management of the patient.

**N M Gupta, MS:** Case chief surgeon, case report concept, manuscript review.

**Kuldeep Dhawan, MS:** Case surgeon, diagnostic work up and plan of surgery, case report design, supervisor.

**Nakul Bansal, MBBS:** Case assistant surgeon, diagnostic work up and case analysis, post-op follow ups, literature and reference search, data collection and data interpretation, original manuscript preparation and preparation of revised drafts.

**Simran Dhawan, MBBS student:** reference search and manuscript revisions.

All authors have read and approved the final manuscript for submission

## Registration of research studies

Not required as this was neither a research study, nor a first-in-man study.

## Guarantor

Dr Kuldeep Dhawan.

## Provenance and peer review

Not commissioned, externally peer-reviewed.

## Declaration of Competing Interest

The authors have no conflict of interests to declare.
